# Environmental concerns on the use of the electrospinning technique for the production of polymeric micro/nanofibers

**DOI:** 10.1038/s41598-024-58936-5

**Published:** 2024-04-09

**Authors:** Angela Malara

**Affiliations:** Department of Civil, Energy, Environment and Material Engineering, University Mediterranea of Reggio Calabria, Via Zehender, Loc. Feo di Vito, 89124 Reggio Calabria, Italy

**Keywords:** Electrospinning, Nanofibers, Environmental impacts, Life cycle assessment, Sustainability, Characterization, Materials science, Nanoscale materials, Techniques and instrumentation

## Abstract

The production of micro and nanofibers through the electrospinning technique is a well assessed technology that finds application in a variety of fields. Indeed, the specific features of electrospun fibers, as well as the possibility to be modelled and functionalized, ensure their great versatility. In the last decades, the widespread use of electrospun fibers promoted studies related to the evaluation of both human health and environmental risks associated to their handling and exposure. However, to date, the environmental impact strictly related to the use of the manufacturing process has been barely considered. Therefore, the present work aims to assess the environmental impacts of the electrospinning technology used to produce micro and nanofibers. To this purpose, a model polymer was systematically electrospun, varying the main system, process and external parameters, that control the electrospinning technique. A simplified life cycle assessment analysis was finally used to evaluate how the fibrous morphology, closely linked to the choice of the technological parameters, intrinsically affected the environmental impacts.

## Introduction

Polymeric nanofibers are thin solid fibers with diameters at the nanoscale, exhibiting distinctive characteristics, among which, significant surface area per unit mass, low weight and porosity need to be acclaimed, since distinguish them from comparable materials lacking nanoscale attributes. These characteristics are determined by both the precursor polymer and the manufacturing conditions^1^. As demonstrated by the scientific literature, nanofibers exhibit a great degree of versatility and find numerous applications across several fields. Micro and nanofibers have been utilized in water filters^2^, storage devices^3,4^, dye-sensitized solar cells^5,6^, lithium/sodium-ion batteries^7^, sensors^8–11^, adsorbent materials^12–17^, catalysis^18,19^, drugs delivery^20,21^ and textiles^22^, just to cite few. As a result, the demand for polymeric nanofibers is high, due to the requirement of cheap raw materials and mass production of these products. Indeed, it has been forecasted that the global market for polymeric nanofibers should grow from $2.2 billion in 2021 to $6.7 billion by 2026 with a compound annual growth rate of 25.1% for the period 2021–2026^23^.

As evidenced, nanomaterials offer numerous technical and commercial opportunities but at the same time can pose risks to the environment and to both human and ecosystem health and safety^24^. Consequently, there has been a growing challenge on the development of sustainable, cost-effective, and environmentally friendly nanomaterials, along with the promotion of green chemistry^25^.

However, it should be considered that not only the potential toxic effects on human health are negative aspects for sustainability, being most of the synthetic polymer products not naturally degradable, raising environmental issues. But, also the high energy consumption for their production, pose social and financial burdens that lead to the generation of undesirable, harmful, and dangerous wastes and, last but not least, the difficulties in managing the end of life in post-treatment processes^26^. The toxicological aspect of nanomaterials has been the most investigated^27–29^, taking into account both human health and environmental risks associated to their handling and exposure. On the other side, very little is known regarded the negative impacts related to the nanotechnologies used for the production of nanomaterials. Thus far, there is a very limited number of studies dealing with the assessment of the sustainability and environment-friendly statement of manufacturing nanotechnologies^30,31^.

There are many ways to fabricate micro and nanofibers, such as self-assembly, melt-blowing, ultrasonically-blowing, solution blowing, electrospinning, and direct blow-spinning^32–35^. Among all the above methods used to produce nanofibers, electrospinning is considered a very effective technique and in the last decades has been widely adopted to produce organic, inorganic and hybrid nanofibers. The success of this method originates in the possibility to generate continuous polymer fibers from the micro- down to the nano-meter size through the use of a simple system that does not demand high temperatures or pressures. Even for these reasons it has been regarded suitable and convenient for the industrial scale up^36^. Therefore, while the advantages of electrospun polymers have been extensively explored, there is a growing need to understand and mitigate the environmental implications associated with their production.

In this view, it is important to proper design, develop and evaluate nanofibers production processes and Life Cycle Assessment (LCA), one of the most important tools used to quantify the environmental impacts, can be used to this purpose. In particular, LCA can be useful for evaluating potential environmental footprints, to compare alternatives, choose production routes and improve processes themselves^37,38^. Furthermore, the impacts of the production process, the use and the disposal or recycling of the material have to be evaluated before any conclusion can be drawn.

Piccinno et al.^37^ presented a comparative LCA study on the production of cellulose nanofibers. Two different methods, traditional and innovative, were compared in order to evaluate the main contributor to the environmental impact, finding that the newly developed technology seemed to be a promising and sustainable alternative production route with respect to the traditional one. It is not obvious that a new technology is necessarily environmental superior to traditional alternatives, since many studies have shown that nanotechnologies, that may seem to be environment friendly, may simply shift the negative impacts outside the analysis boundaries or impact categories^31^. A detailed cradle-to-grave life cycle assessment of nano-biopolymers was used to address the economic and scientific issues related to their production using very different technologies^39^. An LCA evaluation was performed on a model system of nanofiber electrospun for Li-ion battery cathode applications, in order to identify promising development pathways and to point out the significant impacts of preparation methods as well as energy consuming electrospinning subprocesses^40^. A simplified study of the environmental impacts related to the anode materials manufacturing process was also reported^7^.

In this study, aiming to improve the environmental sustainability of the electrospinning technology, a simplified comparative LCA analysis was carried out to identify and evaluate the environmental impacts of the electrospinning parameters, at the laboratory scale. Indeed, despite the challenges associated with scaling the results of a LCA analysis from small-scale laboratories to large-scale operations^41^, laboratory research remains invaluable for a deep understanding of fundamental principles underlying process or products. This knowledge is essential to identify key parameters and mechanisms that drive environmental impacts, and to guide large-scale optimizations. Therefore, the novelty of this study is the correlation between the electrospinning parameters, used to obtain a fibrous morphology, and a selected environmental impact category, the Global Warming Potential (GWP), that quantifies a product or process contribution to greenhouse gas emissions. Indeed, it is essential for researchers to prioritize environmental sustainability from the inception of their ideas. Considering the environmental impact at the forefront of research not only demonstrates ethical responsibility but also ensures a valuable contribution for a more sustainable future.

## Methods

### Electrospinning set-up, parameters and study configuration

Polyvinyl acetate (PVAc) is used as a model polymer and ethanol as its solvent^42^. The choice of this polymer is due to its widespread use in numerous studies dealing with the role of electrospinning parameters. Moreover, it is one of the most investigated polymers in a variety of molecular weights and concentrations, allowing to obtain fibrous morphologies in different conditions with high reproducibility^42–45^. Ethanol was purchased by sigma Aldrich as well as PVAc, that was used with three different molecular weights, 100,000 (L-Mw), 170,000 (M-Mw) and 500,000 (H-Mw).

**Figure 1:**
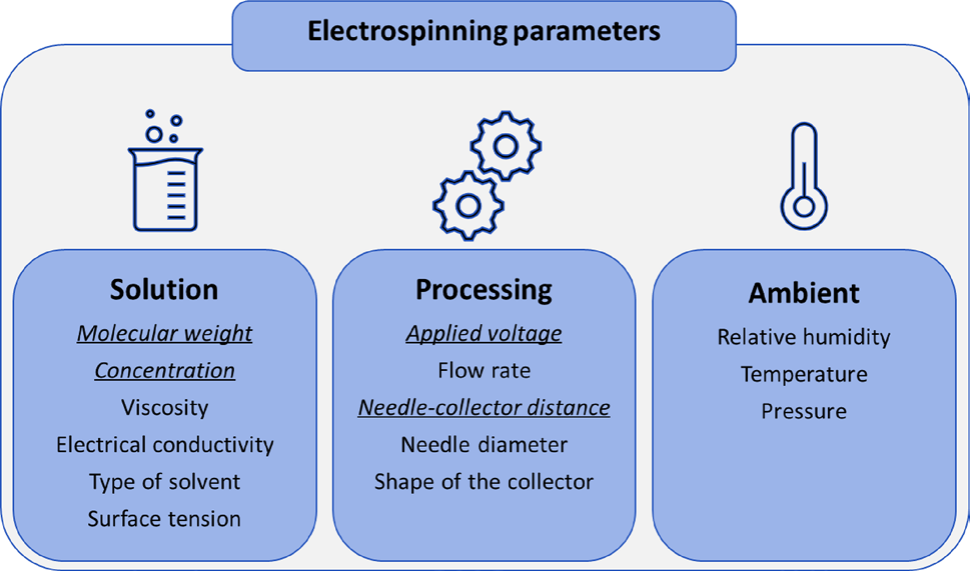
Solution, processing and ambient electrospinning parameters. Underlined parameters are object of the study.

In a typical procedure, PVAc is solubilized in ethanol, magnetically stirred until solubilization (see details in Table 1) and finally electrospun at room temperature (25 °C) and low humidity conditions (40% RH). The polymer solution is then introduced into a glass syringe and pushed through the syringe stainless steel needle by an extruder, able to accurately control the flow rate of the solution. A high-voltage generator is used to apply an electric potential difference between the syringe needle and the copper collector covered by an aluminum foil, so that, if the electrostatic force overcomes the surface tension of the solution drop, a polymer jet is ejected from the so called Taylor cone in a nearly straight line, stretched under the electrostatic field, and finally deposited on the collector in the form of solid nanofibers, thanks to the solvent evaporation^46^. This simple procedure is greatly affected by solution, processing and ambient parameters, schematically reported in Fig. 1.

**Table 1:** LCI for the production of 1 g of polymeric electrospun fibers, at different evaluated conditions.

Material input		Mass (g)	Mass (g)	Mass (g)	Mass (g)	Mass (g)	Mass (g)	Mass (g)
PVAc (L-Mw) 100,000		1.00	–	–	–	–	–	–
PVAc (M-Mw) = 170,000	L-c = 15 wt%	–		–	1.00			
	M-C = 20 wt%		1.00				1.00	1.00
	H-C = 25 wt%					1.00		
PVAc (H-Mw) = 500,000		–	–	1.00	–		–	–
Ethanol		4.00	4.00	4.00	5.60	3.00	4.00	4.00

Non-material input		Energy (kWh)	Energy (kWh)	Energy IkWh)	Energy (kWh)	Energy (kWh)	Energy (kWh)	Energy (kWh)
Phase 1: energy for solubilization		1.2×10^−3^	1.2×10^−3^	1.2×10^−3^	1.2×10^−3^	1.2×10^−3^	1.2×10^−3^	1.2×10^−3^
	Time (h)	2.50	3.00	3.50	2.00	3.50	3.00	3.00
Phase II: energy for electrospinning								
	Time (h)							
	L-d = 10 cm	2.67						
	M-d = 14 cm		2.70		3.70	2.00	2.70	2.70
	H-d = 18 cm			2.74				
Pump		2.50×l0^−2^	2.50×l0^−2^	2.50×l0^−2^	2.50×l0^−2^	2.50×l0^−2^	2.50×l0^−2^	2.50×l0^−2^
Power Supply HV	L-V = 9 kV						4.50×10^−2^	
	M-V = 13 kV	6.50×10^−2^	6.50×10^−2^	6.50×10^−2^	6.50×10^−2^	6.50×10^−2^		
	H-V = 17 kV							8.50×10^−2^
Phase III: energy for drying		–	–	–	–	–	–	–

Air emissions		Mass (g)	Mass (g)	Mass (g)	Mass {g)	Mass (g)	Mass (g)	Mass (g)
Ethanol		4.00	4.00	4.00	5.60	3.00	4.00	4.00

As discussed in literature, the effect of each parameter has been extensively investigated^47^. Briefly, solution parameters play an important role in the fiber formation during the manufacturing process. *Polymer concentration* is critical to obtain smooth fibers with controlled diameters, since ranging from low to high concentrations, electrospray and helix-shaped micro-ribbons can be respectively observed^48^. Similar effects on the morphology of fibers are also due to the *molecular weight*. Additionally, *solution viscosity* is strictly related to both parameters, finally determining the upper and lower boundaries of the electrospinning windows. The *type of solvent* is partially responsible of the solution *surface tension*, together with the *viscosity*, as well as it gives a great contribution to the solution *electrical conductivity* and along with the *polymer type*, it induces a poor or good fiber formation.

Processing parameters are mainly function of the *applied voltage*, that should be higher than the threshold voltage necessary to the charged jets to be ejected from the Taylor cone. However, the affection of the applied voltage on the diameter and morphology of electrospun fibers is a little controversial^49,50^. The *flow rate* takes into account the time the polymeric jet needs to get charged, finally influencing fibers diameter. Specifically, high flow rates promote large droplets formation with a high volume of solution drawn from the needle and consequently long dry time, causing web formation instead of single fibers. On the contrary, low flow rates are more desirable as to give the solvent sufficient time for evaporation^51^. The *shape of the conductive collector* can instead determine the type of fibers alignment and their transferring on different substrates. The *distance* between the collector and the tip of the needle can also affect fibers formation: a long distance could give beads along fibers, whereas a short distance could hinder solvent evaporation, finally resulting in thinner or thicker fibers, respectively. Less influencing is the effect of the *needle diameter*, reported to barely influence fibers dimension^47^. Finally, ambient parameters, as *relative humidity (RH)*, *temperature* and *pressure* can mainly modify the solution viscosity and solvent evaporation rate.

Basically, all the discussed parameters, with countless combination, contribute to fibers formation, morphology and dimension. Considering the cause-effect relationship among some of them, in this study, just an accurate selection of parameters was considered and their multifactor impact on the production process was evaluated. Therefore, to this purpose, ambient conditions were kept constant in all experiments: atmospheric pressure, T = 25 °C and 40% RH, easily achievable and not energetically expensive on a laboratory scale. Nevertheless, even in large-scale productions, where also mild conditions can be energy-intensive, the expenses required to meet the ambient parameters can be neglected because they remain the same across all the production process and hence not included in case of comparative analysis. Once chose the polymer model, PVAc, the type of solvent, ethanol, was fixed too^42^. Moreover, ethanol is considered a green and sustainable solvent at low environmental risk^52^. It is noteworthy that despite the specific selection of PVAc, the study was intended to proof the concept and to be eventually extended to whatever kind of polymer, aside specific considerations and modifications. Among solution parameters, just the molecular weight and the concentration were varied, being all the other parameters strictly related and dependent on these. As regard instead the processing parameters, applied voltage and needle-to-collector distance were studied, having the needle diameter, fixed at 0.7 mm, and the collector shape, squared, poor influence as formerly discussed. In accordance with previously reported results related to electrospun PVAc fibers^16^, the flow rate was set at 1.5 ml/h and kept constant for all the experiments, as to reduce the possible variables. Indeed, the equal flow rate gives the same energetic contribute for the production of each sample, finally irrelevant for the impact evaluation.

**Figure 2:**
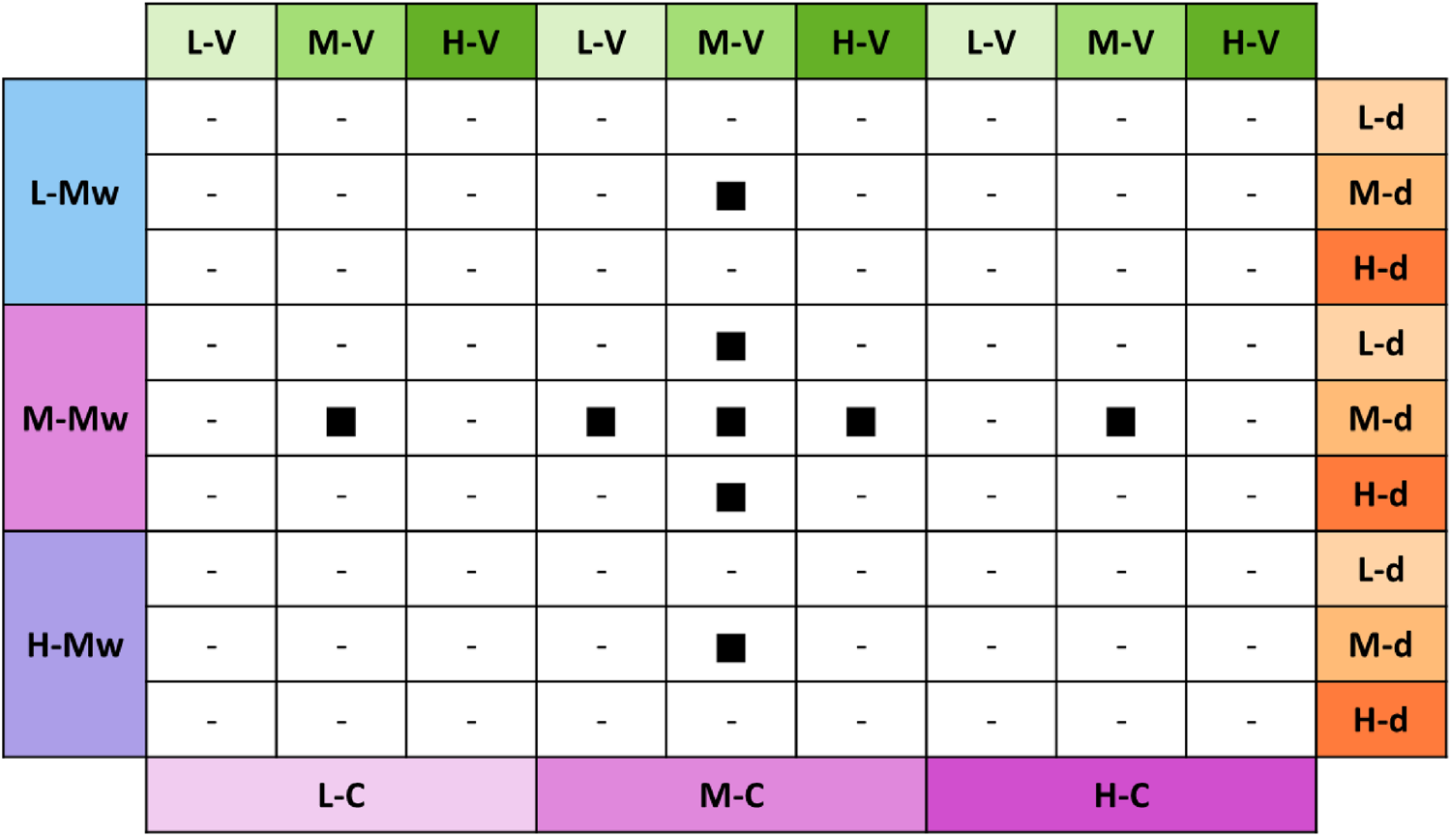
Design of experiments related to the 81 forecasted samples versus electrospinning parameters: polymer molecular weights, polymer concentrations, collector-to-needle distances, and applied voltages. Black squares represent the 9 conducted experiments and codes have the following meaning: L (Low), M (Medium), H (High), Mw (Molecular weight), V (Voltage), d (distance) and C (Concentration).

Following these criteria and constraints, a set of 81 experiments was forecasted. Basically, the use of three various molecular weights for the model polymer, each of which should be tested with at least three different concentrations, was the cause of the significant number of trials that were estimated. Additionally, each combination of molecular weight and concentration required to be electrospun using three different voltages and three different needle-to-collector distances. According to the conventional approach, in order to obtain accurate experimental results, it would have been necessary to carry out all of the forecasted tests. However, following an equally reliable and rigorous scientific methodology, for the purpose of the optimization of trials and the minimization of the environmental impacts proposed in this study, the design of experiment was reduced to 9 tests, able to fully evaluate the impact of the electrospinning production process. The choice of the design of experiment was determined by the orthogonal design^53^, a robust approach that integrates the meticulousness of experimental design with the ability to generate prompt results by examining multiple factors at once. It is regarded as a good tool to avoid randomized trials and yield significantly better outcomes in a more expedient and cost-effective way. Indeed, the method allows to test the effectiveness of many factors simultaneously in a single experiment, potentially revealing their interconnections, with significantly fewer experiments. In Fig. 2 a schematic representation of the forecasted and conducted experiments is reported towards the selected parameters.

Briefly, for each molecular weight three different polymer/solvent concentrations namely low concentration (L-C = 15%), medium concentration (M-C = 20%) and high concentration (H-C = 25%) were tested, in agreement with previous studies^42,44,47^. Solutions were electrospun by applying a voltage between the stainless-steel needle, positively charged, and the grounded collector, a plate of copper usually covered by an aluminum foil. The electric potential difference was applied in the range 9–17 kV (low voltage, L-V = 9 kV, medium voltage M-V = 13 kV and high voltage, H-V = 17 kV) over a collection distance between 10 and 18 cm (low distance, L-d = 10 cm, medium distance M-d = 14 cm and high distance, H–d = 18 cm), at the constant rate of 1.5 ml/h (Electro-spinner 2.0, Linari Engineering s.r.l.). The resulting fibrous mats were dried from the excess of solvent at room temperature. This step was considered to be not energetically wasteful, neither for the laboratory dimension nor for the potential scale up. Moreover, thanks to the process itself, the evaporation of the solvent, depending on its volatility, is generally completed within the deposition time, hence, it should be possible to skip the drying phase as well.

Finally, as to accomplish the aim of the study, the characterization of fibers morphology was carried out by Scanning Electron Microscopy (SEM, Phenom Pro X) over electrospun materials. Fibers diameters distribution was evaluated using the dedicated Fibermetric software, acquiring 200 measures for each sample. The calculated average diameters were reported and considered as absolute reference terms for samples comparison.

### LCA methodology

**Figure 3:**
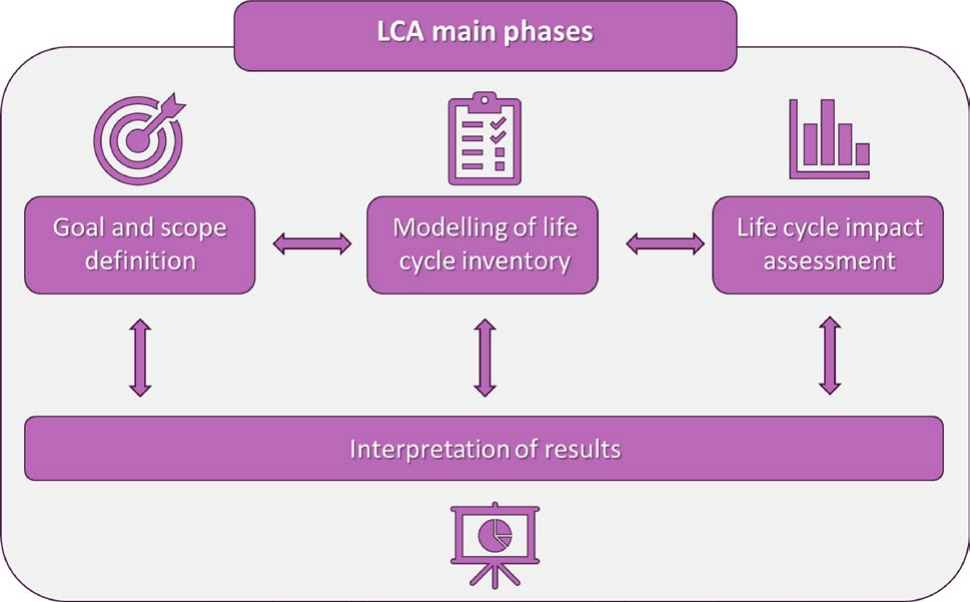
Schematic representation of Life Cycle Assessment main phases.

The LCA method, used to assess the environmental impacts, associated to the electrospinning parameters, on the fibers manufacturing process, was based on the ISO guidelines 14,044 and 14,045^54^. It involves four main critical steps: (a) definition of goal and scope; (b) modelling of life cycle inventory; (c) life cycle impact assessment and (d) interpretation of results^39^, as depicted in Fig. 3.

In the first step, the purpose of the study is defined, together with the boundaries and the limitation for the analysis, that determine the stages of the life cycle to be consider. In addition, the functional unit is also declared as the reference basis to build and model the analysis. The first stage is fundamental to address the LCA study and assess what environmental impact will be considered. In the second stage quantitative information as in-flow of energy, resources, chemicals and out-flow of emission to air, water and land, waste and side products, that are associated which each stage of the study, are collected and modelled. Then, model data are converted to environmental impacts and damage categories, using mid-point or end-point indicators. Specifically, mid-point indicators are directed to reflect the impacts of environment, such as global warming potentials, whereas endpoint, provides insights to decision maker focusing on ecosystem quality, human health and resources. In the final step results are interpretated.

## Experimental

**Figure 4:**
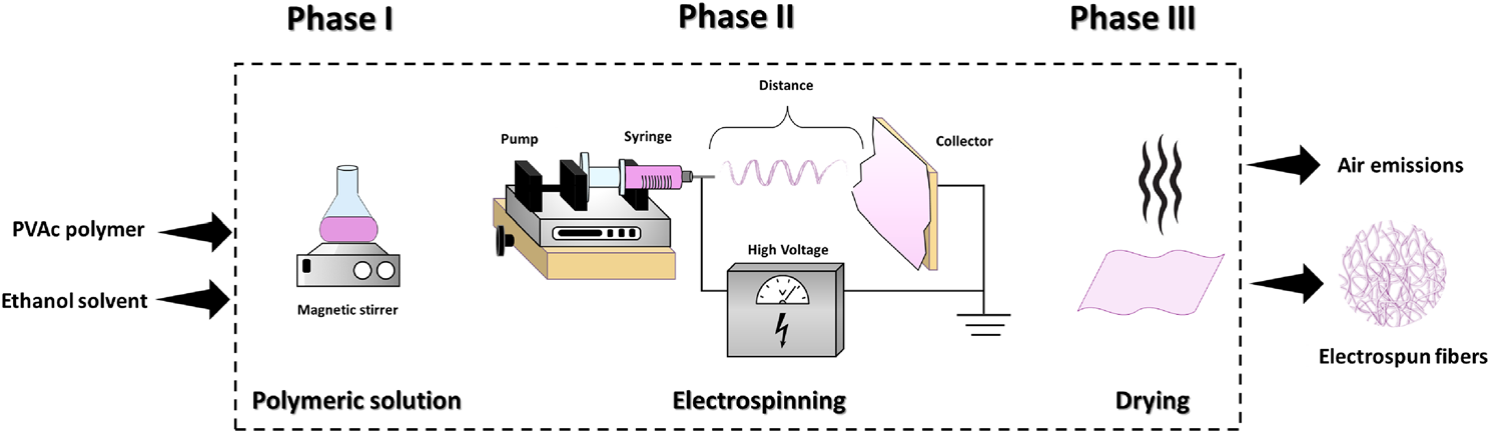
Schematization of the boundaries of the system and identification of phases I, II and III.

A laboratory-scale LCA was approached considering that only experimental data were available. Specifically, being the result of direct measurements, they were considered primary data, in agreement with the regulations. The study was designed as “cradle-to-gate” and the boundaries of the system were defined as depicted in Fig. 4. Those boundaries included three stages: *phase I*, related to the solubilization of the polymer in the solvent; *phase II*, concerning the electrospinning of the polymer solution; and *phase III*, limited to the drying of nanofibers. During the LCA modelling the “cut-off” approach was applied in the case of energy flow related to the extruder, being equal for all the experiments. Similarly, the cut-off rule excluded also external parameters, namely pressure, temperature and relative humidity. Indeed, once they are set, they will give a constant contribute on impacts, that finally can be excluded when comparative LCA analysis is considered.

As regard instead the identification of the functional unit, it should be considered that, in the scientific literature, three levels of complexity can be detected, namely, (i) ‘simple’ functional unit, composed of a single variable; (ii) ‘moderately complex’ functional unit, composed of two variables; and (iii) ‘complex’ functional unit, composed of three or more variables. In this work the function of the system product was “obtaining the fibrous morphology” assuming a “simple” functional unit, without specification about the performance. Using a simple or single-variable functional unit in a system or process offers several advantages compared to employing a moderately complex (two-variable) functional unit in terms of simplicity, cost-effectiveness, reliability, and ease of implementation. For this reason, the functional unit considered was 1 g of polymeric nanofibers produced, useful to offer an easy comparison of different evaluated scenarios.

Successively, once defined the goal, the scope and the functional unit, inventory analysis was carried out, modelling all input and output data considered for the fibers production.

Table 1 shows inventory data related to the production of 1 g of polyvinyl acetate electrospun fibers considering three different molecular weights, three different polymer concentrations, three different applied voltages and three different distances. These data were used for mass balancing and energy consumption evaluation.

Firstly, as provided by LCA analysis, mass balancing was employed to verify the equilibrium between input and output values, facilitating the tracking of material flow throughout the production process. The mass balance was formulated by taking into account the quantities of the incoming reagents, polymer and solvent, as well as the amount of produced fibers in output. Clearly, once fixed the polymer quantity, the amount of solvent employed was directly correlated with the concentration parameter. During the spinning process, the solvent underwent evaporation and released into the atmosphere. Calculations were all related to 1 g of produced nanofibers, as detailed in Table 1.

Subsequently, for each specific production step, the working time was carefully evaluated as to calculate the specific energy consumption. It was considered that different molecular weights or concentrations may require more energy to dissolve the polymer in the solvent. Similarly, the voltage applied during electrospinning greatly affects the energy consumption while conversely, the distance modification has a slight influence, as detailed in the following. The amount of energy consumed during the processing and manufacturing phases were measured for the magnetic stirrer, the power supply and the infusion pump, resulting in accordance with the specific data declared by the instrumentation manufacturers, Heidolph and Linari Engineering, respectively. In detail, measurements were conducted in operational states during the production time. The energy consumption of the magnetic stirrer, set at 800 rpm, had an average value of 1.20⋅10^−3^ kWh, ranging between 2.40⋅10^−3^ and 4.20⋅10^−3^ kW, upon considering the solubilization time from 2.0 to 3.50 h, respectively for the L-C in M-Mw sample and H-C in M-Mw and H-Mw samples. During the process of spinning, the power consumption per hour at 9 kV, 13 kV and 17 kV was measured to be 4.50⋅10^−2^ kWh, 6.50⋅10^−2^ kWh and 8.50⋅10^−2^ kWh respectively. It finally corresponded to a maximum consumption equal to 24.05⋅10^−2^ kW for the sample spun for 3.70 h at M-Mw, L-C, M-d, M-V and a minimum consumption of 12.15⋅10^−2^ kW for the sample spun for 2.0 h at M-Mw, M-C, M-d, L-V. The power consumption of the syringe pump was on average measured to be 2.50⋅10^−2^ kWh during operation. It is noteworthy that the power consumption of the syringe pump remained constant being the infusion rate equal for all the samples. Furthermore, a marginal alteration was noted in the performance of the pump upon using L-Mw and H-Mw or L-C and H-C samples, due the difference in viscosities. Nevertheless, this disparity was so insignificant that it was disregarded and an average value ± 0.02⋅10^−2^ kWh was considered in calculations. The needle-to-collector distance indirectly affected the energy consumption by influencing the variation of the deposition time, closely linked to the applied voltage, and eventually responsible for the different temporary productivity, oscillating between 2.0 and 3.70 h for samples M-Mw, H-C, M-d, M-V and M-Mw, L-C, M-d, M-V respectively. No energy contribution was considered during the drying phase since it took place at room temperature, or eventually skipped as previously discussed.

For the energy and raw materials production processes, the Gabi 6 software integrated with the Gabi Professional and Eco-invent databases were used.

The method selected for the classification and characterization phase of the incoming and outgoing flows of the inventory analysis and for the evaluation was the CML2001, developed by the Institute of Environmental Sciences Leiden University and implemented in the software support tool.

Considering the type of reagents used in the electrospinning process, the impact category applied to quantify the environmental impact of inputs in the whole process of PVAc fibers production was the Global Warming Potential (GWP). Indeed, GWP is frequently given priority because of the pressing need to address climate change. This indicator measures the extent to which a product or process contributes to greenhouse gas emissions, in line with worldwide initiatives to reduce climate impacts. Additionally, GWP enables the comparison of emissions, helping to identify potential solutions and develop strategies for reducing carbon emissions. GWP also provides advantages in terms of transparency and communication, assisting stakeholders in making well-informed decisions. Alongside, other impact categories are also important, but in spite of this, no significant effects were evidenced, considering the burdens of this study and the laboratory scale.

## Results and discussion

**Figure 5:**
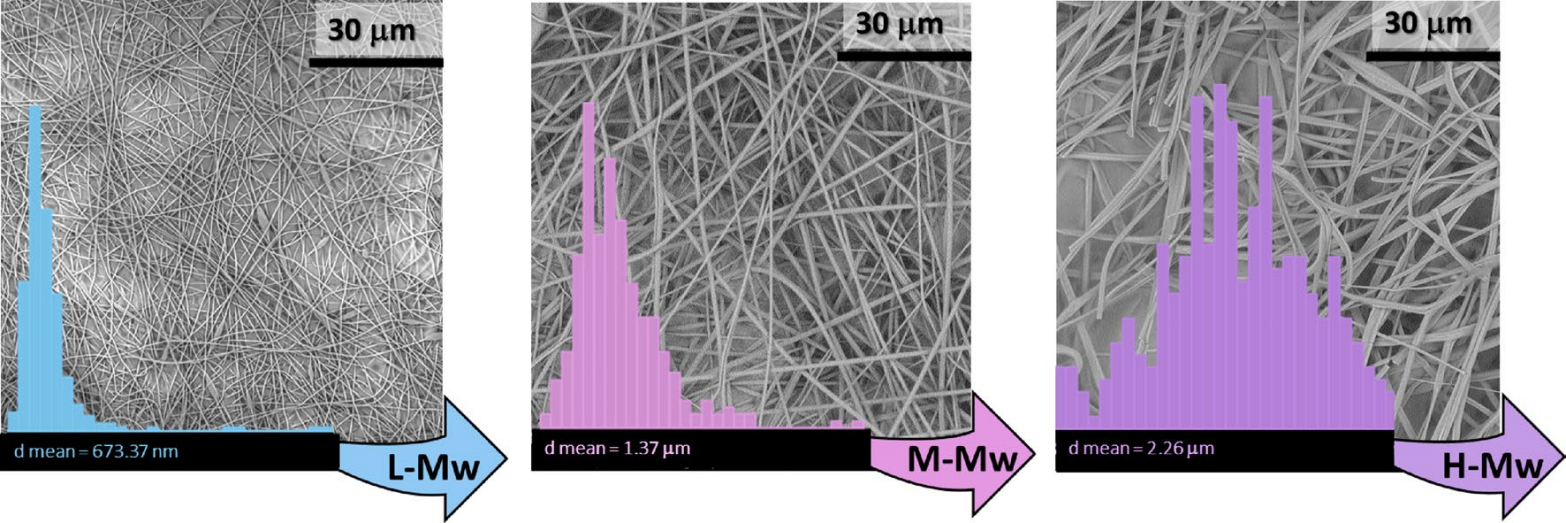
SEM micrographs and mean diameters distribution of fibers electrospun with PVAc at L-Mw (low molecular weight), M-Mw (medium molecular weight) and H-Mw (high molecular weight).

**Figure 6:**
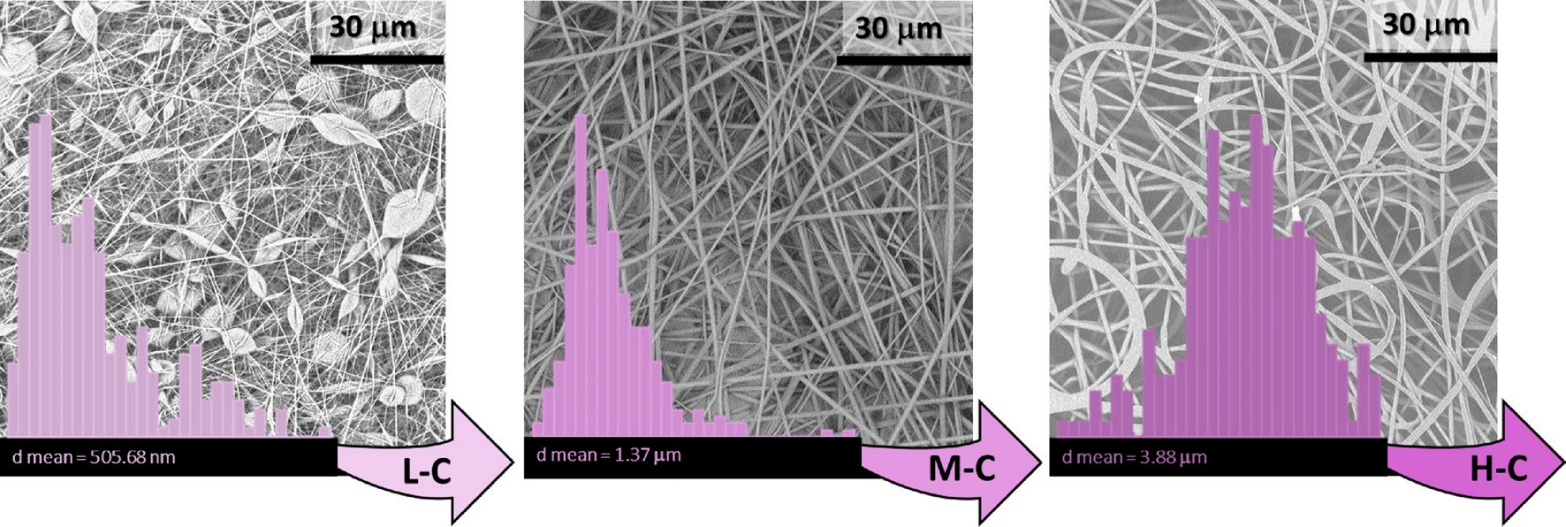
SEM micrographs and mean diameters distribution of PVAc fibers electrospun with increasing weight concentrations L-C, M-C and H-C, namely 15%, 20% and 25%, using the medium molecular weight polymer, M-Mw.

After the systematic modification of the selected electrospinning parameters and the analysis of materials and energy flows, collected electrospun materials were characterized. Both the morphology and the mean diameter of PVAc micro/nanofibers were evaluated by SEM micrographs, aiming to properly correlate the obtained results to environmental impacts assessment. Specifically, as previously discussed, the focus of this study was the correlation between the fibrous morphology obtained as output of the optimized set of trials to one of the most important environmental impact factors, the GWP. This evaluation was individually examined for the chosen electrospinning parameters. In Fig. 5, the effect of the polymer molecular weight was monitored finding that its increment, namely L-Mw, M-Mw and H-Mw, was accountable not only for an increasing in fibers diameters but also for their altered aspect, in analogy to the concentration effect. Indeed, in Fig. 6 it was reported the influence of different polymer concentrations, L-C, M-C and H-C once the medium molecular weight was fixed and adopted. The fast evaporation of the solvent in the jet, together with high polymer molecular weights (see H-Mw) or concentrations (see H-C), appeared as flat ribbon fibers^55^. On the contrary, circular cross-section fibers were generally obtained using both lower molecular weights (see L-Mw and M-Mw) and lower concentrations (see L-C and M-C). However, it is worth nothing that, when concentrations as low as 15% (L-C) in the case of M-Mw PVAc were used, beads formation was favored, proving that the concentration, even in a slight weight percentage variation, was a more sensible parameters with respect to the molecular weight. A similar consideration was also in accordance with the measure of the diameters reported in Figs. 5 and 6, where it was evident that fibers diameters followed a Gaussian distribution. Indeed, mean diameters of samples at different concentrations exhibited a bigger size range (between 505.68 nm and 3.88 μm) compared to samples at different molecular weights, whose values were included between 673.37 nm and 2.26 μm. Moreover, when circular cross-section fibers were electrospun, a narrow diameter distribution was reported, whereas a broader distribution was evidenced in irregular, beaded and flat/ribbon fibers.

**Figure 7:**
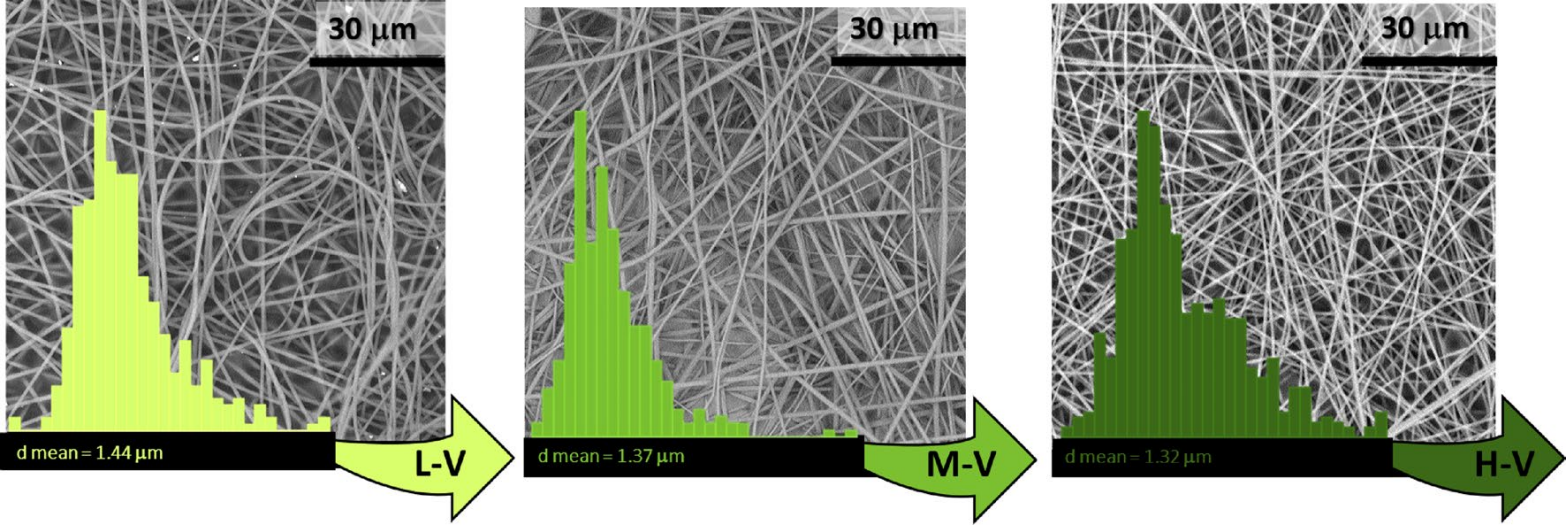
SEM micrographs and mean diameters distribution of fibers electrospun with PVAc at L-V (low voltage), M-V (medium voltage) and H-V (high voltage).

**Figure 8:**
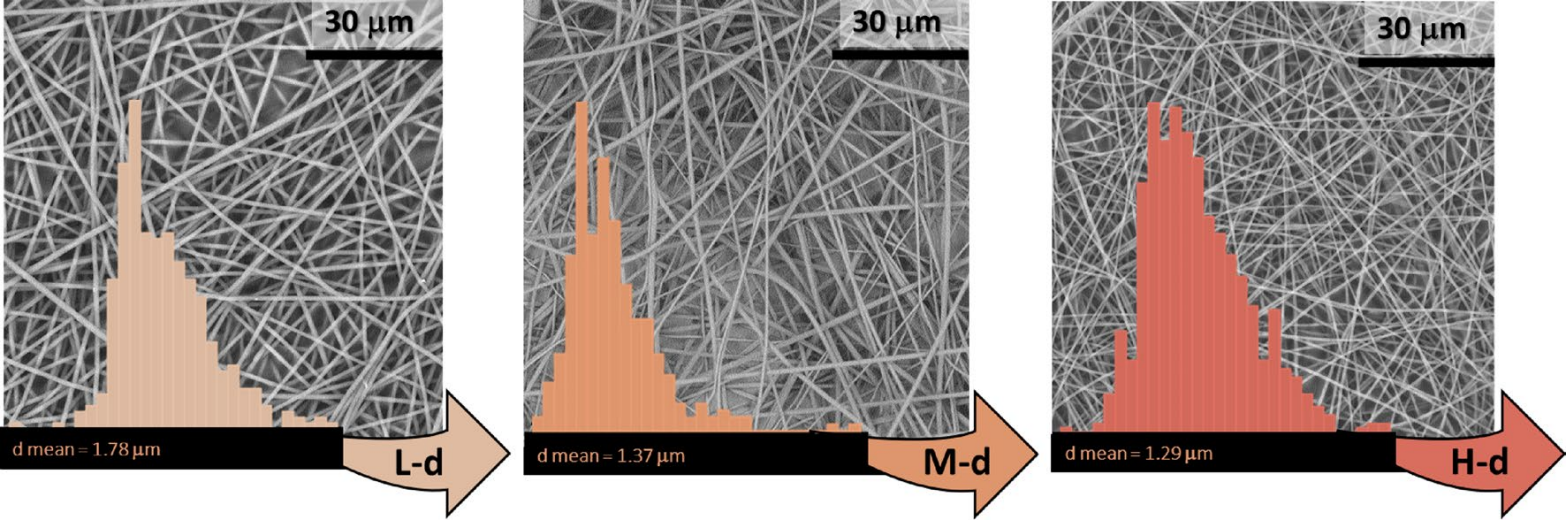
SEM micrographs and mean diameters distribution of fibers electrospun with PVAc at L-d (low distance), M-d (medium distance) and H–d (high distance).

In Fig. 7, SEM micrographs and mean fibres diameter, due to the effect of the applied voltage, are displayed. The morphology of fibres was fully maintained, appearing with smooth surfaces and homogeneous structures, for all the applied voltages. However, with respect to previous parameters, a less marked variation in diameters was obtained, funding that low applied voltages produced bigger fibres diameters and conversely, high values led to lower fibres sizes, also in accordance with other studies^49^. Similarly, the effect of the distance was systematically studied considering three representative values, as depicted by SEM micrographs in Fig. 8. It was found a very low difference upon varying the distance, since it was measured respectively an increase or decrease of mean fibres diameter of barely the 5%. Moreover, as before, homogeneous and smooth fibres were electrospun. Even these fibres diameters displayed a normal distribution, characterized by a narrow width when regular and homogeneous fibres were obtained upon electrospinning parameters variations.

**Figure 9:**
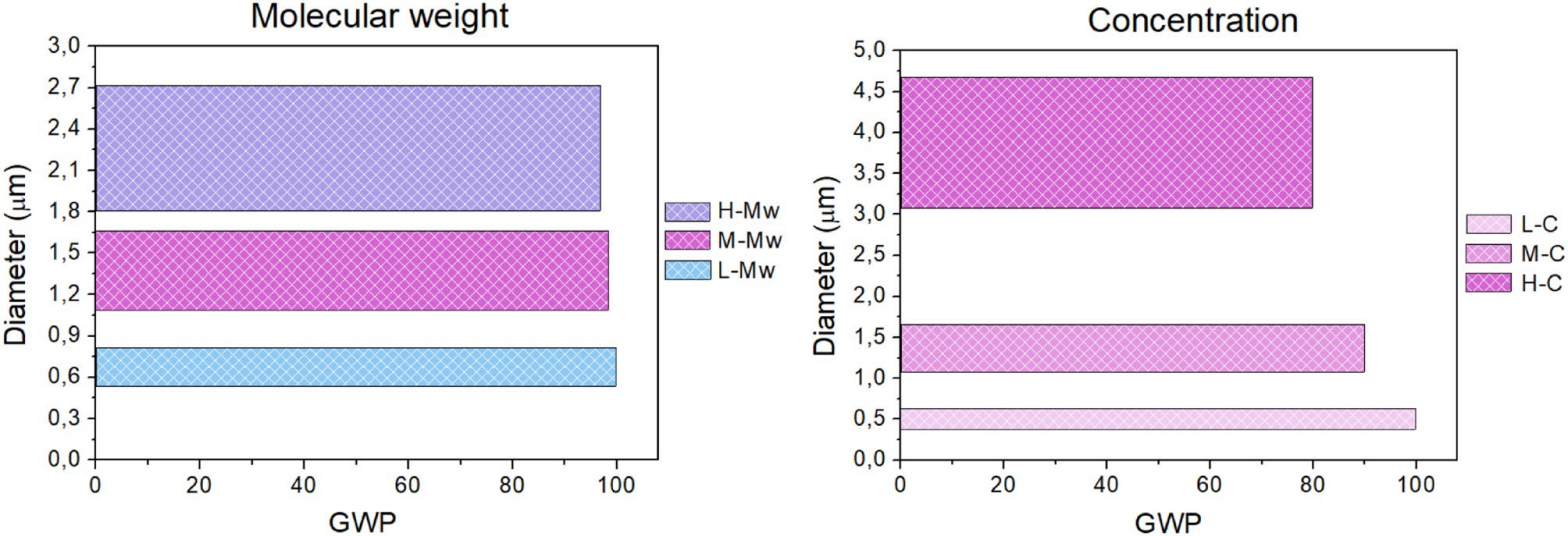
GWP trend versus fibres diameters related to molecular weight and polymer concentration effects.

Through the LCA analysis, the GWP was directly correlated to fibers diameters and reported in Fig. 9, firstly considering system parameters, molecular weights and polymer concentrations. It resulted that the difference in molecular weight affected the assessment of the impacts less than 1%. Indeed, despite the different distribution of diameters, similar GWP values were registered, due to a slight variation in the consumption of the solubilization energy.

As regard polymer concentration, it should be pointed out that using a spinning solution at low concentration a greater GWP impact was obtained. This phenomenon occurs due to the fact that solutions with lower concentrations tend to exhibit lower viscosity, while maintaining the same molecular weight, compared to solutions with higher concentrations. In addition, it should be considered that the solubilization time is reduced for low concentration, due to the higher quantity of solvent and, in order to collect the same amount of nanofibers, a greater quantity of precursor solution needs to be electrospun, taking therefore a longer electrospinning time. As the concentration increases, the GWP decreases and correspondingly the size of fibers diameters also increases.

**Figure 10:**
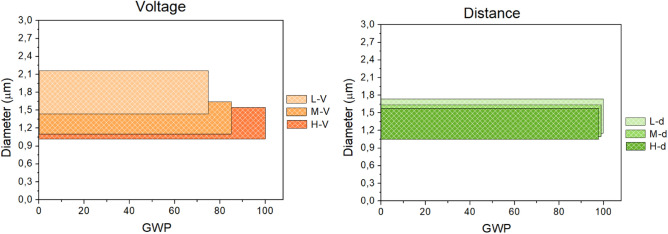
GWP trend versus fibres diameters related to needle-to-collector distance and applied voltage.

The GWP trend versus fiber diameters and related to process parameters (applied voltage and needle-to-collector distance) is reported in Fig. 10.

An increase in the applied voltage leads to an evident increase in the GWP impact, while varying the needle-to-collector distance, keeping constant the potential, does not significantly alter the GWP. As conceivable, results are in agreement with the SEM characterization, where scarce differences arose in fibers diameters when different voltages were used. In particular, fibers size had a narrow variation of the mean diameter considered, confirming the controversial role of applied voltage on the morphology effect^49^. Similar findings were also obtained for the needle-to-collector distance, appearing quite irrelevant on both impact and fibers dimension. Indeed, fibers diameters remained almost unchanged as well as the impact quantification, differing, more or less, of 1%. However, both parameters resulted eventually responsible for the different temporary productivity, being the deposition time different for the specific spinning conditions, as previously discussed and detailed in Table 1.

Among the system parameters examined by this comparative LCA analysis, it was showed a significant dependence of the GWP on polymer concentration, while in the case of process parameters, the applied voltage significantly influences the GWP, that indeed resulted less affected by the needle-to-collector distance and polymer molecular weight.

Therefore, in conclusion, it was possible to optimize the production of electrospun fibers, with a regular fibrous morphology in a nanometer range, when a minimum value of GWP was considered.

Finally, as related instead to the impact of the different phases for the production process, it was quite expected the electrospinning phase to be the most impacting one. In turn, this was essentially due to the lower impact of the other examined phases, as solubilization and drying.

## Conclusion

Although the LCA study was developed on a laboratory scale, important considerations were drawn from this analysis, especially from a qualitative perspective, also relevant for a future scale up. The achievement of the fibrous morphology by the electrospinning technique, was pursued as the focus of this study through the preferential optimization of those parameters that minimized the environmental impacts associated with their single or joint variability. The experimental procedure, based on the organization of a limited number of tests, resulted successful in the optimization of the study, allowing a rigorous, fast and cost-effective method to yield significantly better outcomes, avoiding randomized trials.

The single effect of selected parameters affecting the electrospinning technique on GWP, one of the major environmental impact indicators, was evaluated, highlighting the dependence of this indicator on their modifications. It was found that the GWP was influenced by variations in polymer molecular weights by less than 1%, whereas the GWP was shown to be decreased when polymer concentrations were low, namely at 15%. Similarly, it remained almost unchanged, differing, more or less, of 1% upon varying the needle-to-collector distance and was greatly lower when a low applied voltage, as low as 9 kV was used. At the same time, the GWP minimization in relation to solution parameters lead at first attempt to the formation of flat and big fibers, suggesting that regular and thinner fiber morphology could be obtained maintaining the high concentration but lowering the polymer molecular weight, keeping a low GWP. On account of processing parameters, the goal of a regular fiber morphology was achieved with the lowest GWP value, even if there exist chances of adjustment in the diameter values.

Finally, by detecting which stage in the process had the highest environmental impact, LCA results were used to improve the production process by choosing the optimum conditions to produce nanofibrous morphology, regardless the specific characteristics conveyed to fibres.

This simplified LCA analysis will serve as a valuable instrument for providing recommendations and identifying constraints aimed at further mitigating environmental consequences. This also includes the identification of more environmentally friendly and socially responsible practices.

## Data Availability

The datasets used and/or analysed during the current study will be available from the corresponding author on reasonable request.
